# Endoleak Assessment Using Computational Fluid Dynamics and Image Processing Methods in Stented Abdominal Aortic Aneurysm Models

**DOI:** 10.1155/2016/9567294

**Published:** 2016-08-31

**Authors:** Yueh-Hsun Lu, Karthick Mani, Bivas Panigrahi, Wen-Tang Hsu, Chia-Yuan Chen

**Affiliations:** ^1^Department of Radiology, Taipei City Hospital, Zhongxing Branch, Taipei 103, Taiwan; ^2^National Yang-Ming University, Taipei 112, Taiwan; ^3^Department of Mechanical Engineering, National Cheng Kung University, Tainan 701, Taiwan

## Abstract

Endovascular aortic aneurysm repair (EVAR) is a predominant surgical procedure to reduce the risk of aneurysm rupture in abdominal aortic aneurysm (AAA) patients. Endoleak formation, which eventually requires additional surgical reoperation, is a major EVAR complication. Understanding the etiology and evolution of endoleak from the hemodynamic perspective is crucial to advancing the current posttreatments for AAA patients who underwent EVAR. Therefore, a comprehensive flow assessment was performed to investigate the relationship between endoleak and its surrounding pathological flow fields through computational fluid dynamics and image processing. Six patient-specific models were reconstructed, and the associated hemodynamics in these models was quantified three-dimensionally to calculate wall stress. To provide a high degree of clinical relevance, the mechanical stress distribution calculated from the models was compared with the endoleak positions identified from the computed tomography images of patients through a series of imaging processing methods. An endoleak possibly forms in a location with high local wall stress. An improved stent graft (SG) structure is conceived accordingly by increasing the mechanical strength of the SG at peak wall stress locations. The presented analytical paradigm, as well as numerical analysis using patient-specific models, may be extended to other common human cardiovascular surgeries.

## 1. Introduction

Patients with abdominal aortic aneurysm (AAA) have a significantly high risk of suffering from destructive aneurysm rupture, which is a major source of morbidity and mortality in human [1]. AAA affects 8.8% of the population over the age of 65 [2]. This condition has an 11% risk of rupturing, and this rate exponentially increases when the transverse aneurysmal diameter exceeds 5 cm [3]. To prevent sudden aneurysm rupture, the mean enlargement rate and the occurrence of sudden change in the size of AAA are also important check points for subsequent follow-up medical care [4]. AAA is characterized by the degradation of elastinous constituents, the adaptive growth and remolding of collagen, the loss of smooth muscle cells with thinning of the medial wall, the infiltration of lymphocytes and macrophages, and neovascularization [5–8]. The major root cause of AAA is unclear, but abnormal flow-induced mechanical stress that is acting on the vessel wall in blood circulation is a known critical factor that contributes to AAA formation and pathological evolution [8, 9]. An in-depth investigation from the hemodynamic aspect can therefore provide invaluable insights to unveil the convoluted factors that lead to AAA formation and propagation.

Patients aged over 60 years with an aneurysm diameter exceeding 5.5 cm are commonly advised to undergo endovascular aneurysm repair (EVAR) or open AAA repair; however, these patients should be anesthetically and medically prepared for the procedures [10]. Clinical trials show that EVAR reduces the 30-day operative mortality of patients with large AAA by two-thirds compared with open AAA repair [10]. EVAR could be an ideal alternative for AAA treatment, but long-term clinical results are necessary to support the validity of EVAR. In EVAR, a stent graft (SG) is guided from the femoral artery to the aneurysm bulge to shield the aneurysm from the blood flow. This SG can serve as a blood flow conduit through the aneurysm sac [11] to eliminate the blood in the intrasac of aneurysm and therefore reduce the risk of aneurysm rupture [12]. However, the complexity of hemodynamics and biomechanics in the aneurysm region may cause several complications in post-EVAR patients; such complications include an aneurysm expansion and rupture even in a successful EVAR [11], blood seepage into the cavity between the aneurysm wall and the SG wall (termed as endoleak) [12], SG migration, and SG failure [13].

Endoleak formation is associated with the failure of SG implantation and is used as an endpoint in clinical trials [14]. Endoleaks can be divided into five types based on the source of blood flow into the intrasac of aneurysm [11]. Type I endoleaks have a blood flow that originates from a SG attachment site, including the aortic neck and distal iliac attachment sites [15]. With this type, separation occurs between the SG and the native arterial wall, and the direction communication between the aneurysm sac and the systemic circulation is created; this condition can lead to SG failure [11]. Type II endoleaks are those in which blood flows into the aneurysm sac in the retrograde direction through the branch from the portion of the aorta that has not received a SG. Typical sources of Type II endoleaks include the inferior mesenteric and lumbar arteries [11]. Type III endoleaks occur when the structure of a SG fails [15]. Type IV endoleaks are caused by graft porosity and usually identified on the completion of angiography during implantation when the patient is fully anticoagulated [15]. Aneurysm expansion without endoleaks is referred to as endotension or a Type V endoleak [11]. However, whether or not Type I endoleaks are associated with a continued risk of aneurysm rupture and require immediate attention of medical treatments remains controversial [16]. Accordingly, the current study focused on Type I endoleaks to provide direct clinical impacts.

Computed tomographic angiography (CTA) is widely used to detect endoleaks because of the high sensitivity and specificity of this method [17]. However, CTA is less effective than conventional angiography in classifying endoleak types because of the difficulty in determining the direction of a blood flow from a routine CTA process. Another concern for CTA imaging is radiation exposure. High radiation doses of CTA administered during EVAR pose a potential risk of radiation-induced skin damage and later malignancy [18]. Patients who underwent EVAR should detect the damage early to prevent additional endoleak formation. Early endoleak detection reduces the need for unremitting follow-up through CTA examination and thus minimizes radiation exposure. The current study employed a three-dimensional (3D) computational fluid dynamics (CFD) method combined with a series of image processing methods to assess endoleak formation from the hemodynamic perspective.

A high degree of physiological relevance is necessary for a CFD analysis to simulate human cardiovascular flows with high accuracy. In addition to the physiological consistency of the imposed boundary and initial conditions, a direct match of the vascular geometrical features between numerical modeling and patient data is required. Papaharilaou et al. (2007) evaluated the AAA wall stress in an anatomically identical 3D patient-specific AAA model. They used 3D reconstruction software and developed the patient-specific AAA model from the CT images [19]. A similar concept was also adopted to create patient-specific AAA models for in vitro flow dynamic assessment and the wall stress evaluation through an experimental flow visualization technique [8]. Li and Kleinstreuer (2006) studied the effect of Type I endoleak on the stented AAA model through a numerical simulation technique. Their study reveals that there is an increase in the cavity pressure due to the endoleak which increases the probability of aneurysm rupture [20]. The consequences of various types of endoleak in a silicon rubber based AAA model were experimented on by Lu et al. under the maintained physiological conditions, and their study reveals that the presence of endoleak elevates the mean sac pressure to the aortic pressure. These patient-specific models provide useful insight towards the pathological aspect of AAA under the influence of endoleak. Though many researchers highlight the effect of endoleak on the aneurysm, very few researches have been reported till date which can predict either position or formation of the endoleak in post-EVAR patients [21]. To address this issue, as a first step, in the present work, six patient-specific models were constructed from the patients' CT images in order to provide a global view on the relationship between endoleak and its surrounding flow to draw a conclusion on identifying possible endoleak position with a high degree of statistical significance based on hemodynamics. Furthermore, the compliance nature of the aneurysm wall should be considered in AAA flow modeling to study the hemodynamics in AAA patients [22–25]. A fluid-structure interaction (FSI) method was developed to couple the wall effect into the simulation of AAA hemodynamics. This method was first implemented in AAA modeling in 2001 [22], where the complex mechanical interaction between blood flow and wall dynamics in a 3D custom model of an AAA patient was calculated through the FSI method. Several researchers also validated the importance of incorporating the FSI method compared with simple computational models using finite element modeling [23, 25, 26] in the AAA simulation. The FSI method underestimates the peak stress by 9% [23], which can increase to 12.5% if only a homogeneous pressure finite element model is used [19]. An open debate currently exists on whether or not a compliant wall should be considered in fluid dynamic experiments and simulations of AAAs [27]. The coupling of complete FSI in hemodynamic simulations provides a new insight into the examination of AAA-related complications, such as intraluminal thrombus [19], and its relationship with flows in the AAA lumen. In the present work, the FSI method was selected to provide a comprehensive flow assessment with high accuracy for blood dynamic flow behavior in SG.

## 2. Materials and Methods

### 2.1. Clinical Summary

Clinical information of six patients was included in this study. All the patients were of old age (81.5 ± 9.04 years) and had a history of atherosclerosis. Maximum diameter of the aneurysm sac for all the patients was found to be 6.96 ± 1.39 cm. Moreover, dynamic CT angiography scan suggests the presence of either Type I or Type II endoleak. CT scans were available for all the patients and were acquired at Taipei Veterans General Hospital (Taipei, Taiwan) through a CT scanner (Aquilion 64, Toshiba, Tokyo, Japan) with a 64-slice imaging capability and each scanned image had a voxel size of 5 mm in all the spatial directions. Each patient received 120 cc of contrast material before scanning via injection at a rate of 4.5 cc/s. This dose was administered into the cubital vein of the right arm. A bolus-tracking technique was applied in an arterial phase at 1.5 mm intervals for image recoding.

### 2.2. 3D Patient-Specific Anatomical Models

**Figure 1:**
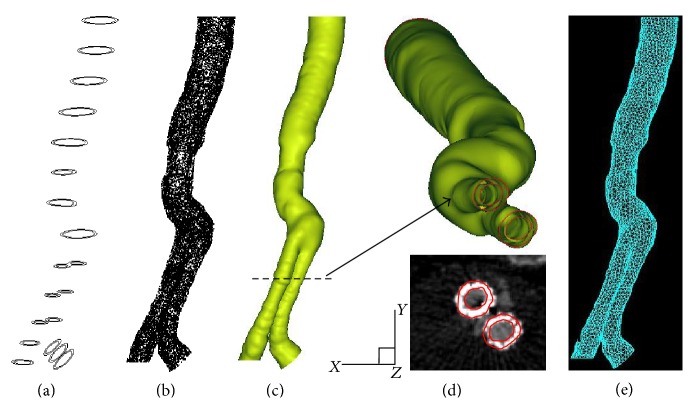
Snapshots of critical steps in 3D reconstruction of a patient-specific AAA with SG model. (a) Lumen segmentation; (b) geometry interpolation; (c) surface spline fitting; (d) selected reconstructed SG features and the corresponding in-plane CT slice in the region of interest; and (e) finite element meshing for CFD analysis.

To provide hemodynamic assessment results with a high degree of clinical relevance, six computational models with patient-specific SG geometry were reconstructed from CT images of AAA patients with SG implants. A series of image processing methods were employed for the reconstruction and smoothing of the 3D surface. In particular, three software programs, namely, Simpleware (Simpleware Ltd., Exeter, UK), Geomagic (Geomagic Inc., NC, USA), and Pro/Engineer (Parametric Technology Corp., MA, USA), were used for the 3D reconstruction as well as smoothing surface of the patient-specific models. CT images were segmented through an intensity threshold method to extract the geometrical features of the implanted SG. Then, image smoothing using a recursive Gaussian filter was conducted to further filter background noise. A 3D anatomical model was subsequently produced in stereolithography format, in which a mesh of triangles was applied to form the shell of the reconstructed SG. Once the 3D model of the SG was constructed, a surface refinement algorithm was applied, and the surfaces and boundaries of the model were subsequently created through the nonuniform rational basis spline method. Detailed 3D reconstruction can be found in the literature [8, 9, 21, 28].  Figure 1 shows a selected schematic of the reconstructed AAA with SG model. In the present work, the patient data were all acquired a few months after SG implantation. Fully grown connective cells and tissues attached to the implants. Therefore, the SG was assumed to have a similar material property to the aneurysm wall. The SG wall was constructed by circumferentially dilating the SG perimeter outward; this wall was modeled as a hyperelastic homogeneous incompressible isotropic material with a uniform thickness of 2 mm [19], a density of 2000 kg/m^3^, Young's modulus of 2.7 MPa, and a Poisson ratio of 0.45 [29].

### 2.3. Computational Fluid Dynamics (CFD) Analysis

The FSI method was coupled with the incompressible Navier–Stokes equations to provide detailed flow patterns, specifically for fluid flows in the SG vicinity and enhance the accuracy of stress distribution calculation on the SG wall structure. In the FSI simulation, fluid forces (blood) were applied onto the structure (SG), and then the structural deformation changed the fluid domain. Dominant fluid variables (pressure and velocity) and wall displacement were selected as solution variables of the fluid flow [30]. The imposing boundary and initial flow conditions were assumed to be identical for the six patient-specific models to provide a systemic investigation unveiling the interactions between the geometrical feature of the implanted SG and the encompassed blood flow dynamics. Blood pulsatility and SG properties were also determined in a similar manner. Blood was treated as a homogeneous, incompressible, and Newtonian fluid with a dynamic viscosity of 0.004 Pa s and a density of 1,055 kg/m^3^ [29]. Physiologically representative inflow velocity and outflow pressure waveforms were applied in the modeling with a pulse period of 1.2 s [12], and the Reynolds number (*Re*) was set as 2234 [9, 31]. To demonstrate grid independence, cell* Re* was calculated, and a convergence was reached for the approximate element number of 155,000. The total element number selected in the modeling for each patient slightly varied because of the different geometric natures of all tested patients. Commercially available software Adina (Adina, Watertown, MA) was used for finite element analysis. The von-Mises stress (wall stress) distribution was calculated and analyzed for each simulation case to represent the complex stress distribution in the wall of each virtual AAA. The von-Mises stress was derived from the distortion energy used in studies of material failure and was calculated from the six components of the stress tensor [32]. Studying the von-Mises stress allows significant interpretation of the hemodynamic flow impact to the SG structure, as evidenced by a previous study where the wall stress was several orders of magnitude larger than the wall shear stress in AAA models [1].

### 2.4. Image Processing of CT Images for Quantification of Endoleak Geometry

**Figure 2:**
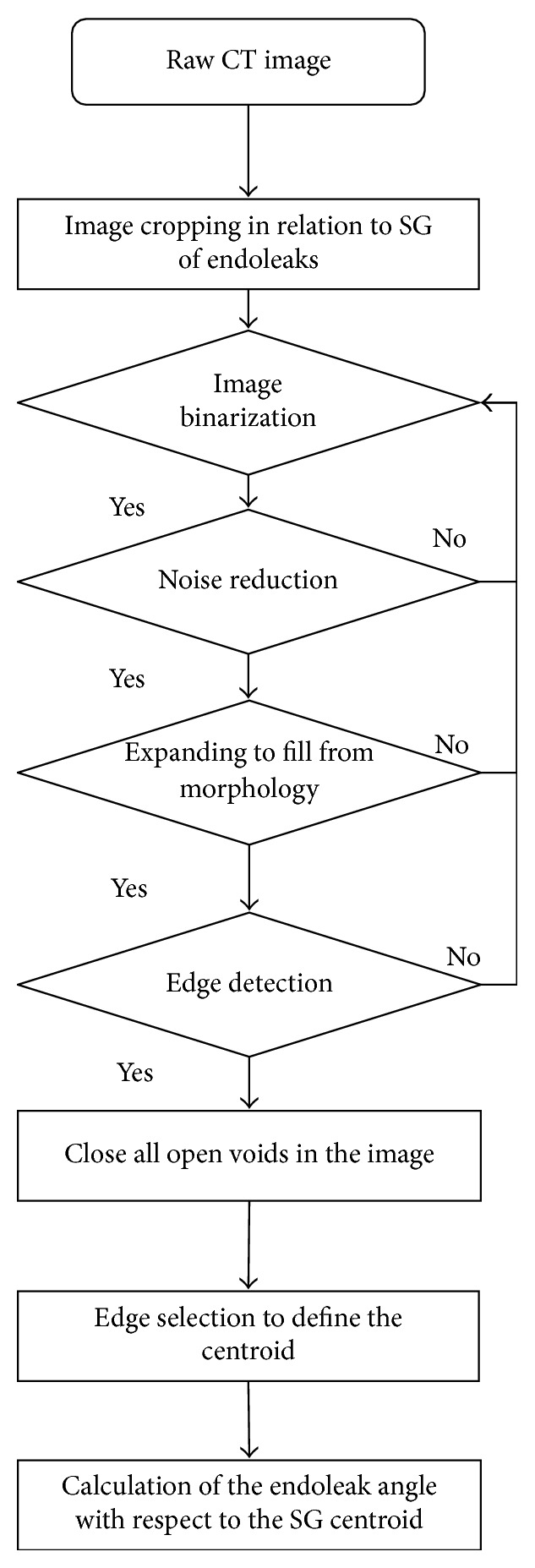
Illustration of the applied image processing algorithms describing the process flow for endoleak geometrical characterization.

A series of image processing steps was employed to characterize the geometrical features of endoleak in AAA with SG-implanted models. Endoleak positions, with respect to the SG centroid, were quantified through the presented image processing flow. The results are shown in Figure 2. In addition to image binarization, region-of-interest (ROI) cropping from the raw CT image was performed. Cropping segmentation was performed to enhance and isolate the ROI. This step was followed by a series of image filtering techniques, such as noise reduction to sharpen the edges and ROI deblurring. Noise reduction is crucial to avoid artifacts and preserve anatomical details. This operation was achieved by replacing each pixel with the average pixels in a square window surrounding this pixel. Canny edge detection was performed through a 2D spatial gradient measurement on an image, and regions that corresponded to edges were highlighted. This method is usually applied to determine the approximate absolute gradient magnitude at each point in an imported grayscale image. After the edges of endoleaks and SG structures were located, an image morphological operation was applied to close all voids in the ROI. Subsequently, the centroids of each segmented structure were calculated. The entire process was conducted with an in-house imaging processing Matlab (MathWorks, Natick, MA, USA) programming code.

### 2.5. Matching Index

**Figure 3:**
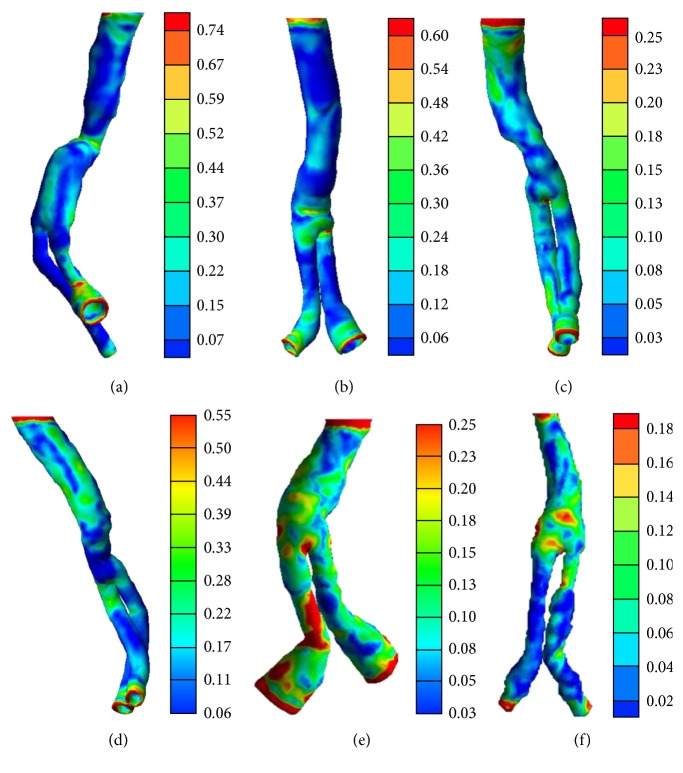
Wall stress distribution of six patient-specific models. (a)–(f) correspond to models I–VI, respectively. The color bar unit is in MPa.

**Figure 4:**
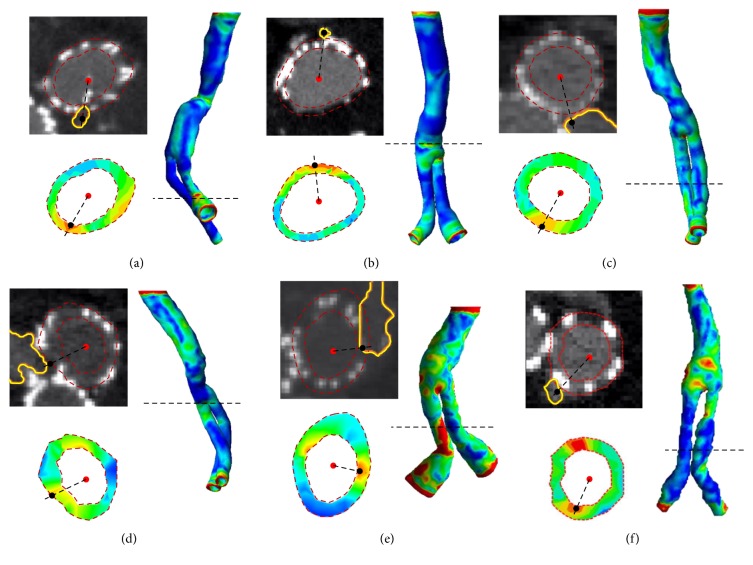
Location comparison between the endoleak (CT image) and the wall stress peak (color 3D and slicing plots) of six patient-specific models. Panels (a)–(f) correspond to models I–VI, respectively. SG and endoleak boundaries were outlined as red dotted and yellow solid lines, respectively. Each slice plot was extracted from the corresponding 3D plot at the level outlined as a black dashed line.

Six patient-specific models of AAA with SG were reconstructed carefully because of their high geometrical irregularity. The wall stress distribution along the height of each SG structure was obtained through FSI calculation. To provide significant clinical relevance, endoleak CT image slices were selected and compared with the corresponding wall stress distribution slices to determine the possible correlation between the endoleak and the local wall stress peak. Slices with location matching between the wall stress peak and the endoleak appearance on the SG structure are highlighted in Figures 3 and 4. The degree of the reported location agreement was further quantified and presented as a matching index using

(1) Matching  index%=AngleEndoleak  to  SGC−AngleWs  to  SGCAngleWs  to  SGC,

where SGC denotes the centroid of SG.

## 3. Results and Discussion

### 3.1. Wall Stress Distribution in Patient-Specific Models

The wall stress distribution on the six patient-specific models was calculated (Figure 3). The distribution was complex with large regional variations along the length of each model. In general, the peak wall stress was concentrated in regions with relatively high degrees of kinking and curved bifurcations, as well as in the vicinity of the model inlet and outlets. These findings well agreed with the results of the previously published researches where higher wall shear stress was evidenced in these aforementioned regions [12, 33]. The maximum calculated wall stress ranged from 0.81 MPa in model I to 0.2 MPa in model VI because of variations in the model structure even under identical imposed boundary conditions. Moreover, the calculated wall shear stress value has a magnitude similarity with the results of previous researches [20], where the authors have evidenced an elevated wall stress of 0.3 MPa near the bifurcating point of the SG model. In model I, the local maximum wall stress was located at the posterior wall of a daughter branch region. The peak magnitude was comparable with that close to the branch outlet and was relatively larger than that in the necking region (anterior region) of the SG. Notably, this stress peak appeared only in one daughter branch. This result indicates that the stress distribution is not symmetrical in both daughter branches. The symmetric outlet pressure boundary conditions were applied to both branches; thus, this asymmetrical wall stress distribution was contributed mainly by the SG geometry differences between them. Further analysis of the wall distribution in model II showed that the peaks were concentrated not only on the SG inlet and outlets but also on the anterior wall of the bifurcation. The peak wall stress was calculated up to 0.66 MPa. The distributed wall stress is concentrated in these aforementioned locations; hence, additional attention should be given to these regions in terms of mechanical strength reinforcement of the SG. Consistent wall stress distribution was found in models III, IV, V, and VI. Aside from the wall stress concentrated on the previously referred locations, local peak wall stress was also found along the length of one daughter branch in model V. This result can be attributed to the fact that the SG was significantly oriented out of the plane in the posterior end of this daughter branch with respect to the anterior side. The flow was diverted significantly, which resulted in high local wall stress distribution due to the pronounced flow impingement on the posterior wall.

### 3.2. Correlation between Endoleak and Peak Wall Stress through Location Comparison

 Li and Kleinstreuer (2006) [20] computationally simulated the type I endoleak in a stented AAA model and indicated that the presence of endoleak maximizes the EVG wall shear stress near the bifurcating point while reducing its magnitude, which certainly differs to our hypothesis that there is an elevation in the EVG wall shear stress corresponding to endoleak position. To delve into possible mechanism underlying the onset of endoleak formation, the location of endoleak and peak wall stress were quantified. Thus, each patient CT image slice that matched the peak wall stress location from the corresponding 3D simulation model was reviewed and quantified through image processing. The obtained endoleak position was compared with the peak wall stress location, and the results are shown in Figure 4. In Figure 4(a), where the first clinical patient data and the modeling results are shown, an endoleak appeared on the distal region of the SG. We measured from the 2D CT image that the endoleak (outlined in yellow) was located at 265° with respect to the SG daughter branch centroid (indicated as red solid dot). This location was very close to the 230.9° wall stress peak with respect to the SG daughter branch centroid in the modeling results. This location agreement (with a matching index of 14.8%) reveals a possible correlation between endoleak formation and the locally concentrated wall stress. However, more than one wall stress peaks were found on the wall stress distribution slice. Another wall stress peak was located on the right hand side of the SG centroid with no endoleak appearance. These results suggest that endoleak formation may possibly occur when the local wall stress is high.

Further investigation of the second patient data set shows that endoleak location agrees well with the peak wall stress location, with a matching index of only 4.4% (Figure 4(b)). Inconsistent with the previous first patient data set where the endoleak was identified in one of the daughter branch regions, the endoleak appeared before the apex of the branches on the second patient data set. A narrowing SG diameter was found at the local geometry in the surrounding endoleak area. This distorted geometry may increase the local wall stress and subsequently induce endoleak formation because of the high mechanical loading acting on the SG structure. Patient-specific boundary conditions, such as the local hydrodynamics and SG strength, should be considered to verify the convoluted effects that contribute to endoleak formation. The third patient data set is shown in Figure 4(c). The exact leaking site from the SG is difficult to identify in the CT image because the endoleak spread over both SG branches. Alternatively, the middle point of the endoleak boundary that was in contact with both SG branch boundaries was selected as the leaking site. The matching index was calculated to be 12.5%, which is still within the acceptable range. Similar endoleak geometry was found with the boundary elongated through both daughter branches of the SG (Figure 4(d)). The middle point was again identified, and its location was compared with that of the wall stress peak of a SG daughter branch. The matching index in this case was measured to be 5.2%, which shows a high degree of position matching between the endoleak and the wall stress peak. The fifth patient data set is shown in Figure 4(e) with a position match occurring in the SG daughter branch between the endoleak and the wall stress peak. Specifically, the endoleak site was identified by an experienced radiologist (the leading author of the presented study) through examination of the sequential CT slices. A secondary wall stress peak again appeared on the opposite side of the investigated wall stress peak site. However, no endoleak was found in the vicinity of this secondary wall stress peak. Therefore, the matching index was calculated on the wall stress peak that was close to the verified endoleak with a value of 9.4%. Another patient data set is shown in Figure 4(f), where both the endoleak and the wall stress peak were found in one of the daughter branches of the SG. The matching index in this case was calculated to be 6.0%, indicating a high matching degree between the locations of the endoleak and the wall stress peak. The patient data sets suggest that a good agreement in location exists between the endoleak appearance and the local wall stress peak.

Table 1 presents a summarized position comparison evaluated through the matching index calculation from the six patient data sets. The conclusive points of view are described in the following. First, the calculated matching index values from these data sets were in a high level of agreement ranging from 4.4% to 14.8%. These consistent results support the idea that endoleak formation occurs because of the local high wall stress contributed by the local flow and SG structure interactions. This finding can be beneficial for a better SG design to reduce the chances of a secondary incision caused by the failure of previously implanted SG. The effects of radiation exposure during the typical long-term follow-up care using CT imaging can therefore be omitted. However, intensive investigation should focus on patient-specific hemodynamics. The precision of the obtained results can be increased by considering the flow boundary conditions of patients in modeling the wall stress analysis. Second, endoleak formation may occur in the daughter branches of the SG, where the wall stress is usually high because of the distorted geometry in these two branches. Therefore, the SG design phase should be given a major consideration to reduce the possibility of endoleak formation. Third, a significant barrier was posed while searching for the exact leaking point from the SG for the endoleak angle calculation. A well-trained radiologist or clinic physician should assist with the endoleak identification. An efficient imaging-based method that can capture the dynamic blood flow behavior nonintrusively is necessary to accurately identify the endoleak position. Moreover, the image processing steps, such as image segmentation and 3D reconstruction, are labor intensive. Thus, future advances in automatic imaging software development are warranted to reduce processing time and human errors in this regard. Experimental flow visualization tools, such as particle image velocimetry, can be used to provide a validation test case as a follow-up study in revealing the causes of endoleak formation.

### 3.3. Limitations

A major limitation to the current study is that mechanical properties of thrombus were not taken into account during the computational analysis. An aneurysm thrombus load substantially affects the hemodynamics inside the stent graft and may develop endoleak [34, 35]. Still, these biomechanical properties vary from person to person making it difficult to consider these properties for computation which were therefore excluded. Additional studies in this aspect will be carried out once the aforementioned issue is resolved.

## 4. Conclusions

The matching index values in the six patient data sets were all calculated to be lower than 15%. This finding indicates a high degree of correlation between the locations of the endoleak and the local wall stress. This investigation was achieved by performing a series of imaging processing methods to analyze the CT images of patients in a nonintrusive manner in conjunction with patient-specific models for wall stress calculation. The presented analytical paradigm is reliable and robust with high clinical relevance. The results of this study may be used as a basis for future improvement in terms of SG designs to reduce the possibility of SG reoperations. Despite some limitations, the presented technique can be extended to other local hemodynamics of interest.

## Figures and Tables

**Table 1:** Summary of the position correlation between the endoleak and the local wall stress peak through the presentation of the matching index calculation.

	Angle of endoleak to SGC	Angle of WS to SGC	Matching index (%)
Model I	265.1	230.9	14.8
Model II	87.8	91.9	4.4
Model III	275.2	244.7	12.5
Model IV	203.1	214.3	5.2
Model V	8.5	336.9	9.4
Model VI	234.6	249.7	6.0

## References

[1] Li Z.-Y., Sadat U., U-King-Im J. (2010). Association between aneurysm shoulder stress and abdominal aortic aneurysm expansion: A Longitudinal Follow-Up Study. *Circulation*.

[2] Newman A. B., Arnold A. M., Burke G. L., O'Leary D. H., Manolio T. A. (2001). Cardiovascular disease and mortality in older adults with small abdominal aortic aneurysms detected by ultrasonography: the cardiovascular health study. *Annals of Internal Medicine*.

[3] Reed L. W. W., Hallett J. W., Damiano M. A., Ballard D. J. (1997). Learning from the last ultrasound—a population-based study of patients with abdominal aortic aneurysm. *Archives of Internal Medicine*.

[4] Sterpetti A. V., Schultz R. D., Feldhaus R. J., Cheng S. E., Peetz D. J. (1987). Factors influencing enlargement rate of small abdominal aortic aneurysms. *Journal of Surgical Research*.

[5] López-Candales A., Holmes D. R., Liao S., Scott M. J., Wickline S. A., Thompson R. W. (1997). Decreased vascular smooth muscle cell density in medial degeneration of human abdominal aortic aneurysms. *American Journal of Pathology*.

[6] Ailawadi G., Eliason J. L., Upchurch G. R. (2003). Current concepts in the pathogenesis of abdominal aortic aneurysm. *Journal of Vascular Surgery*.

[7] Watton P. N., Raberger N. B., Holzapfel G. A., Ventikos Y. (2009). Coupling the hemodynamic environment to the evolution of cerebral aneurysms: computational framework and numerical examples. *Journal of Biomechanical Engineering*.

[8] Chen C.-Y., Anton R., Hung M.-Y. (2014). Effect of intraluminal thrombus on patient-specific abdominal aortic aneurysm hemodynamics via stereoscopic PIV and CFD modeling. *Journal of Biomechanical Engineering*.

[9] Anton R., Chen C. Y., Hung M. Y., Finol E. A., Pekkan K. (2014). Experimental and computational investigation of the patient-specific abdominal aortic aneurysm pressure field. *Computer Methods in Biomechanics and Biomedical Engineering*.

[10] Greenhalgh R. M., Brown L. C., Kwong G. P. (2004). Comparison of endovascular aneurysm repair with open repair in patients with abdominal aortic aneurysm (EVAR trial 1), 30-day operative mortality results: randomised controlled tria. *The Lancet*.

[11] Stavropoulos S. W., Charagundla S. R. (2007). Imaging techniques for detection and management of endoleaks after endovascular aortic aneurysm repair. *Radiology*.

[12] Li Z., Kleinstreuer C. (2006). Analysis of biomechanical factors affecting stent-graft migration in an abdominal aortic aneurysm model. *Journal of Biomechanics*.

[13] Charonko J., Karri S., Schmieg J., Prabhu S., Vlachos P. (2009). *In vitro*, time-resolved PIV comparison of the effect of stent design on wall shear stress. *Annals of Biomedical Engineering*.

[14] Desai M., Eaton-Evans J., Hillery C. (2010). AAA stent-grafts: past problems and future prospects. *Annals of Biomedical Engineering*.

[15] Heikkinen M. A., Arko F. R., Zarins C. K. (2004). What is the significance of endoleaks and endotension. *Surgical Clinics of North America*.

[16] Lawrence-Brown M. M. M. D., Sun Z., Semmens J. B., Liffman K., Sutalo I. D., Hartley D. B. (2009). Type II endoleaks: when is intervention indicated and what is the index of suspicion for types I or III?. *Journal of Endovascular Therapy*.

[17] Rozenblit A. M., Patlas M., Rosenbaum A. T. (2003). Detection of endoleaks after endovascular repair of abdominal aortic aneurysm: value of unenhanced and delayed helical CT acquisitions. *Radiology*.

[18] Weerakkody R. A., Walsh S. R., Cousins C., Goldstone K. E., Tang T. Y., Gaunt M. E. (2008). Radiation exposure during endovascular aneurysm repair. *British Journal of Surgery*.

[19] Papaharilaou Y., Ekaterinaris J. A., Manousaki E., Katsamouris A. N. (2007). A decoupled fluid structure approach for estimating wall stress in abdominal aortic aneurysms. *Journal of Biomechanics*.

[20] Li Z., Kleinstreuer C. (2006). Effects of major endoleaks on a stented abdominal aortic aneurysm. *Journal of Biomechanical Engineering*.

[21] Lu Y.-H., Chen C.-Y., Menon P. G., Liu K.-T., Lin H.-H. (2014). Hemodynamic effects of endoleak formation in abdominal aortic aneurysm patients with stent-graft implants. *Journal of Medical and Biological Engineering*.

[22] Di Martino E. S., Guadagni G., Fumero A. (2001). Fluid-structure interaction within realistic three-dimensional models of the aneurysmatic aorta as a guidance to assess the risk of rupture of the aneurysm. *Medical Engineering and Physics*.

[23] Scotti C. M., Shkolnik A. D., Muluk S. C., Finol E. A. (2005). Fluid-structure interaction in abdominal aortic aneurysms: effects of asymmetry and wall thickness. *BioMedical Engineering Online*.

[24] Scotti C. M., Jimenez J., Muluk S. C., Finol E. A. (2008). Wall stress and flow dynamics in abdominal aortic aneurysms: finite element analysis vs. fluid-structure interaction. *Computer Methods in Biomechanics and Biomedical Engineering*.

[25] Fraser K. H., Li M.-X., Lee W. T., Easson W. J., Hoskins P. R. (2009). Fluid-structure interaction in axially symmetric models of abdominal aortic aneurysms. *Proceedings of the Institution of Mechanical Engineers, Part H: Journal of Engineering in Medicine*.

[26] Scotti C. M., Finol E. A. (2007). Compliant biomechanics of abdominal aortic aneurysms: A Fluid-Structure Interaction Study. *Computers and Structures*.

[27] Biasetti J., Gasser T. C., Auer M., Hedin U., Labruto F. (2010). Hemodynamics of the normal aorta compared to fusiform and saccular abdominal aortic aneurysms with emphasis on a potential thrombus formation mechanism. *Annals of Biomedical Engineering*.

[28] Lara M., Chen C.-Y., Mannor P. (2011). Hemodynamics of the hepatic venous three-vessel confluences using particle image velocimetry. *Annals of Biomedical Engineering*.

[29] Leung J. H., Wright A. R., Cheshire N. (2006). Fluid structure interaction of patient specific abdominal aortic aneurisms: a comparison with solid stress models. *BioMedical Engineering Online*.

[30] Bathe K.-J., Zhang H., Ji S. H. (1999). Finite element analysis of fluid flows fully coupled with structural interactions. *Computers and Structures*.

[31] Chen C., Antón R., Hung M., Menon P., Finol E. A., Pekkan K. (2014). Effects of intraluminal thrombus on patient-specific abdominal aortic aneurysm hemodynamics via stereoscopic particle image velocity and computational fluid dynamics modeling. *Journal of Biomechanical Engineering*.

[32] Menon P. G., Teslovich N., Chen C.-Y., Undar A., Pekkan K. (2013). Characterization of neonatal aortic cannula jet flow regimes for improved cardiopulmonary bypass. *Journal of Biomechanics*.

[33] Li Z., Kleinstreuer C. (2005). Blood flow and structure interactions in a stented abdominal aortic aneurysm model. *Medical Engineering and Physics*.

[34] Sampaio S. M., Panneton J. M., Mozes G. I. (2004). Proximal type I endoleak after endovascular abdominal aortic aneurysm repair: predictive factors. *Annals of Vascular Surgery*.

[35] Armon M. P., Yusuf S. W., Whitaker S. C., Gregson R. H. S., Wenham P. W., Hopkinson B. R. (1998). Thrombus distribution and changes in aneurysm size following endovascular aortic aneurysm repair. *European Journal of Vascular and Endovascular Surgery*.

